# Analysis of Candidate Genes in Occurrence and Growth of Colorectal Adenomas

**DOI:** 10.1155/2009/306786

**Published:** 2009-10-29

**Authors:** Sylviane Olschwang, Déwi Vernerey, Vanessa Cottet, Alexandre Pariente, Bernard Nalet, Jacques Lafon, Jean Faivre, Pierre Laurent-Puig, Claire Bonithon-Kopp, Catherine Bonaiti-Pellié

**Affiliations:** ^1^Centre de Recherche en Cancérologie de Marseille, INSERM U891, 13009 Marseille, France; ^2^Institut Paoli-Calmettes, Department of Biopathology, 13009 Marseille, France; ^3^INSERM, U535, 94800 Villejuif, France; ^4^Université Paris-Sud, 94800 Villejuif, France; ^5^INSERM, U866, 21000 Dijon, France; ^6^Centre Hospitalier, Gastroenterology Unit, 64000 Pau, France; ^7^Centre Hospitalier, Gastroenterology Unit, 26200 Montélimar, France; ^8^Centre Hospitalier, Gastroenterology Unit, 13080 Aix-en-Provence, France; ^9^Centre Hospitalier Universitaire, 21000 Dijon, France; ^10^Université Paris-Descartes, INSERM U775, 75005 Paris, France

## Abstract

Predisposition to sporadic colorectal tumours is influenced by genes with minor phenotypic effects. A case-control study was set up on 295 patients treated for a large adenoma matched with polyp-free individuals on gender, age, and geographic origin in a 1 : 2 proportion. A second group of 302 patients treated for a small adenoma was also characterized to distinguish effects on adenoma occurrence and growth. We focussed the study on 38 single nucleotide polymorphisms (SNPs) encompassing 14 genes involved in colorectal carcinogenesis. Effect of SNPs was tested using unconditional logistic regression. Comparisons were made for haplotypes within a given gene and for biologically relevant genes combinations using the combination test. The *APC* p.Glu1317Gly variant appeared to influence the adenoma growth (*P* = .04, exact test) but not its occurrence. This result needs to be replicated and genome-wide association studies may be necessary to fully identify low-penetrance alleles involved in early stages of colorectal tumorigenesis.

## 1. Introduction

Colorectal cancer (CRC) is one of the most common human malignancies in Western countries. The majority of the cases develop from a premalignant lesion, the adenomatous polyp [1]. Colorectal adenomas have high malignancy potential when they are large in diameter and/or present with severe dysplasia and/or a villous component [2]. Colonoscopic polypectomy has been documented to significantly reduce the incidence of colorectal cancer [3, 4]. Therefore, the identification of factors associated with the development of colorectal adenoma represents a major goal in colorectal cancer prevention. They could indeed allow the selection of individuals at risk of CRC who may benefit from a screening by colonoscopy.

The adenoma-carcinoma sequence suggests that colorectal adenomas and adenocarcinomas share common environmental and genetic risk factors. An increased risk of colorectal tumors has been found in relatives of patients with large adenomas [5, 6]. A case-control study had suggested that family history of colorectal cancer influenced only the growth of adenomas or their malignant transformation [7]. However, relatively few epidemiologic studies explored genetic risk factors in colorectal adenomas.

We investigated, through a case-control study, the relation between polymorphisms within a series of candidate genes involved in colorectal tumorigenesis and putatively in the formation and the development of colorectal adenomas such carcinogen metabolism enzymes, methylation enzymes, DNA repair genes, oncogenes and tumor suppressor genes [8].

## 2. Materials and Methods

### 2.1. Constitution of the Patients and Control Groups

The GEnetics of ADEnomas (GEADE) study is a case-control and family study of patients with high-risk adenomas (≥10 mm) [9]. The data were obtained from 18 participating gastroenterology units of general hospitals in France. From September 1995 to March 2000, 306 consecutive patients with newly diagnosed colorectal large adenoma (LA) were enrolled in the study. Patients with personal cancer history, familial adenomatous polyposis, established hereditary nonpolyposis colorectal cancer or inflammatory bowel disease were excluded. To distinguish genetic factors involved in the occurrence of adenomas or in their growth, 307 cases with small adenomas (with a diameter smaller than 0.5 cm) (SA) and 572 polyp-free controls (with normal colonoscopy) (PF) were simultaneously enrolled in the same units. All patients and controls were of Caucasian origin. Reason for referral, family history of CRC, completeness of colonoscopy were registered for all patients and controls. Two PF per LA cases were selected as controls within over 2000 PF for matching on age, gender, and geographic area. Patients with SA were relatively rare and could not be matched with LA cases.

Blood specimens were obtained at time of colonoscopy and those patients who presented with a polyp were included only when histological examination revealed the adenomatous nature of the lesion. As polyps were totally removed during colonoscopy, their natural evolution could not be scored. After longitudinally section, half of the tumor material was fixed for histologic analysis and half was frozen for molecular characterization. Twenty individuals had to be excluded because of insufficient tumor material: 11 patients with LA, 5 with SA, and 4 PF. The final groups contained 295 patients with LA, 302 with SA, and 568 PF as controls. Details of these groups have been reported by Lièvre et al. [10]. All patients and controls signed an informed consent after approval of the study by an ethic committee for biomedical research (Le Kremlin-Bicêtre) and the database was declared to the national committee Commission Nationale de l’Informatique et des Libertés (CNIL).

### 2.2. Genes Studied and Genotyping Procedure

Genes have been selected for their role in colorectal tumorigenesis and for the presence of frequent neutral polymorphisms. The polymorphisms of *MMP1*, *MMP3,* and *MMP7* gene promoters, that are responsible for the degradation of extracellular matrix components, had been previously studied and were not considered in the present analysis [10]. Three genes, *TS*, *UGT1A1,* and *MDR1*, are implicated in folates, bilirubin metabolism, and transport of xenobiotic respectively. All these pathways are suspected to play a role in cancer occurrence or in the transformation of benign lesions, folates by interfering with DNA synthesis and repair, bilirubin by its known antioxydant properties, and transport of xenobiotic by its detoxication function. Case-control reports [8] exemplified the relevance of these three genes in addition to *HRAS1*. Three additional pathways have been secondary studied including the WNT pathway (*APC, CTNNB1, AXIN2*), the p53 pathway (*TP53, BAX*), the TGFB pathway (*TGFBR2*), the main MMR and BER genes (*MSH2, MLH1, MYH*), and *CARD15* as part of the family with LRR domains. All these genes have been strongly assessed for their major role in oncogenesis, cycle cell control, apoptosis regulation, cell adhesion and migration, proliferation, DNA repair as their main functions.

Genotyping has been performed according to the nature of the polymorphisms. Single nucleotide polymorphisms (SNPs) have been characterized by Taqman analysis. The VNTR of HRAS1 was studied by conventional electrophoresis after PCR amplification on 1.2% agarose gels at 2 volts/cm for 15–18 hours.

### 2.3. Statistical Analysis

Hardy-Weinberg proportions were tested for each polymorphism. Linkage disequilibrium (LD) between pairwise loci was estimated using the *D*′ [11] and *r* ^2^ [12] measures. Association was first tested for each polymorphism separately. Genotype-specific odds ratio (OR) and 95% confidence intervals (CIs) were computed using unconditional logistic regression adjusted on matching factors; Wald test was used to assess the global effect of each polymorphism. The homozygous genotype for the more frequent allele among controls was set as the reference class. Homogeneity of allele frequencies within geographic regions was previously checked. Tests of homogeneity and unconditional logistic regression were done using the SAS package software. The association was further examined using the combination test, that allows the analysis of all possible combinations of SNPs within a given gene or tightly linked genes to test their association with the disease [13]. For each SNPs combination, the method computes a statistic test contrasting the genotypic (or haplotypic) distribution between cases and controls. Because all these tests are correlated (many of them are nested in each other and the SNPs are likely to be in LD), a permutation procedure has been implemented which displays a significance level adequately adjusted for multiple testing. First, we used the FAMHAP12 software to apply this method by performing haplotypic tests [14]. Then the COMBINTEST (Jannot, personal communication) was used to perform genotypic tests. The method was extended to the test of combined polymorphisms on different genes that may act interactively. To avoid multiple tests biases, we chose a three-step strategy that minimizes the number of comparisons. First, we considered two-by-two combinations of polymorphisms chosen on their plausibility to interact with each other, for instance, *APC* and *MYH* both responsible, when mutated, for adenomatous polyposis. The second step consisted in considering all combinations within a same pathway. Finally, as a combination of polymorphisms may have an effect even if the genes are not suspected to interact with each other and are not involved in the same pathway, all possible combinations were considered if the first two steps did not reveal any significant association. When several combinations of polymorphisms were significant within a given test, it was possible to test nested combinations using Chi-square calculation to determine whether a given polymorphism adds a significant contribution to the association found [14].

## 3. Results

The average age was very similar in patients (62 years for LA and 61 years for SA) and in PF controls (61 years). The sex ratio (male/female) was quite similar in LA (1.7) and in PF controls (1.5), slightly lower in SA (1.2) because of the absence of matching.

Table 1 describes the characteristics of the polymorphisms studied and the allele frequencies in controls. The distribution of genotypes in controls was consistent with Hardy-Weinberg proportions for all polymorphisms except for *TP53.3* (*P* = .0013 without correction for multiple testing). A similar departure from H-W proportions (*P* = .010) was found in the HapMap European population (http://www.ncbi.nlm.nih.gov/projects/SNP/) for this polymorphism.

All polymorphisms within a same gene were moderately or not found in LD except *MYH*; *MYH.1* and *MYH.2* are in quasicomplete disequilibrium (*D*′ = 1 and *R* ^2^ = 0.99). As these two polymorphisms appeared to be equivalent, we analysed only *MYH.1*.

Analysis of single polymorphisms for the different comparisons, LA versus PF, LA versus SA, and SA versus PF did not reveal any association for most of the genes studied. Table 2 indicates the *P*-values obtained for each polymorphism. The *P*-values found to be less than .05 were obtained for *APC.1, MDR1.2, *and* AXIN2.9*. The corresponding OR and 95% confidence intervals are given in Table 3.

The results of the haplotypic and genotypic combination tests performed for each gene or gene combination are shown in Table 4. When considering each gene separately, only *APC* displayed a significant difference between small and large adenomas (*P* = .014 in haplotypic analysis and *P* = .07 in genotypic analysis). As *APC.1* alone and the combination *APC.1-APC.2* appeared both significant, we tested whether *APC.2* provided a significant contribution to the association, which was not the case (*χ* ^2^ = 2.12, 1df) showing that the association was solely due to the *APC.1* (p.Glu1317Gln) polymorphism. Logistic regression indicated an odds ratio of 7.95 (CI: 1.05–354.3). The combination *APC-MYH* appeared also significant with a global *P*-value of .045. However, the only significant combination was *APC.1-APC.2* already found, showing that *MYH* does not provide any additional contribution to the association between *APC* and adenomas. No difference was found for the other two comparisons, that is, SA versus PF and LA versus PF. Finally, the analysis of all possible combinations did not provide any significant result.

## 4. Discussion

We investigated the role of candidate gene polymorphisms in a case-control study of patients with large adenomatous polyps (*n* = 295), compared to patients with small adenomatous polyps (*n* = 302) and polyp-free controls (*n* = 568). The 38 polymorphisms studied belonged to 14 genes possibly involved in increasing of colorectal cancer risk [8]. The reason for comparing these different groups of patients was that different genes might be involved in the different steps of carcinogenesis [7]. Using this case-control study, we had shown that *MMP* polymorphism was most probably involved in the occurrence, but not in the growth of polyps [10]. Using the combination test, we had shown that the effect was due to a specific combination of *MMP1-MMP3* polymorphisms which was not found using logistic regression. Indeed, a major property of this test is that it allows the detection of polymorphisms with low marginal effects [13].

Multiple testing may be a limitation of studies in which several polymorphisms in several genes are tested. The combination test allows a correction for multiple tests on tightly linked markers, which other methods of correction such as Bonferroni, Benjamini-Hochberg, or FDR (false discovery rate) do not. In the present study, we could show that the positive results obtained for *AXIN2* and *MDR1* when analyzing single polymorphisms were false positives as these results were not confirmed when using the combination test. For independent markers, there was no need for correcting for multiple testing as our results were essentially negative.

Our study did not give evidence for an effect of any of the gene polymorphisms studied except the p.Glu1317Gly variant of the *APC* gene. This variant was already described in the previous studies with apparently conflicting results. Some studies reported an effect of this variant in patients with multiple adenomas or colorectal cancer [15–17], and some others found an effect in adenomas but not in cancer [18, 19]. More recently, Hahnloser et al. [20] reported an effect of this variant in a group of patients with a small number of adenomas (1 to 3 lifetime adenomas) and in a group of CRC cases. None of these studies considered the size of adenomas and could differentiate adenomas at high-risk from those at low-risk of cancer. Our results favour the hypothesis that the p.Glu1317Gly variant would influence the growth and not the occurrence of adenomas, and would have thus indirectly an influence on colorectal cancer risk. Nevertheless, although the three groups are paired on gender and geographic origin, it is not stated if other known risk factors would influence adenoma growth. It is thus important to plan a specific multivariate analysis when building a replication sample.

Regarding the *TS* polymorphisms, we did not confirm the effect of *TS* suggested in some studies. Chen et al. [21] found a significant effect of the 3R allele of *TS.2*, particularly of the 3R/3R genotype (OR = 3.3). In our study, the OR associated with 3R/3R genotype was 1.43 (CI 0.94–2.16) for LA versus PF and 1.10 (CI 0.68–1.78) for LA versus SA. On the other hand, Ulrich et al. [22] found no marginal effect with any of these two polymorphisms but found a significant interaction of *TS.2* with folate intake. Similarly, the most recent studies [23, 24] found no marginal effect of either polymorphism but a slight interaction with folate or vitamin B6 intake. The groups' sizes in our study were of the same order of magnitude as in the study of Chen et al. [21], but the three other studies which did not show any marginal effect included larger groups (more than 500 individuals in each group). Given the size of our population, we could a priori detect an OR of 1.7 with a power of at least 80%. It is highly probable that the *TS* gene, which produces a key enzyme in folate metabolism, has an effect only in interaction with dietary exposures, which would explain why this effect was not found in our study. Moreover a new SNP has been recently identified in the second repeat of the 3R allele leading to split the allele 3R in 3RC and 3RG alleles as a tri-allelic polymorphism. This variant could explain these discrepant results since patients with 3RC/3RC genotype have a transcriptional activity of TS comparable to those with 2R/2R genotype [25].

In our study, the problem of multiple testing was adequately controlled for each test performed by the permutation procedure implemented in the combination test. In the last analysis step, the tremendous number of comparisons resulted in a drastic correction of each *P*-value, which considerably lowered the testing power. Thus, our negative results certainly do not definitively demonstrate the absence of specific combination influencing colorectal tumorigenesis, but more probably that, if it exists, this (of these) effect(s) is (are) low and could be found through the initial (and further) genome-wide association studies of colorectal cancer testing much larger numbers of patients and controls [26, 27].

## Figures and Tables

**Table 1:** Characteristics of the polymorphisms and minor allele frequencies (MAF) in polyp-free controls.

Gene	Polymorphism	Type	MAF
*TGFBR2*	*TGFBR2.1*	Intronic; A/C (rs11924422)	0.38
	*TGFBR2.2*	Intronic; C/G (rs12493607)	0.34
	*TGFBR2.3*	Intronic; C/G (rs877572)	0.49

*MLH1*	*MLH1.1*	5′UTR; A/G (rs1800734)	0.21
	*MLH1.2*	Genomic; C/T (rs6789043)	0.47

*MSH2*	*MSH2.1*	Intronic; A/G (rs3924917)	0.34
	*MSH2.2*	Intronic; A/G (rs2347794)	0.36
	*MSH2.3*	Intronic; G/T (rs3732182)	0.25
	*MSH2.4*	Genomic; C/T (rs11125135)	0.22

*MYH*	*MYH.1*	coding, missense; C/G (rs3219489)	0.25
	*MYH.2*	Intronic; C/T (rs2185549)	0.25

*TP53*	*P53.1*	5′UTR; A/G (rs2909430)	0.11
	*P53.2*	Coding, missense; C/G (rs1042522)	0.23
	*P53.3*	Intronic; C/G (rs8064946)	0.11

*AXIN2*	*AXIN2.1*	3′UTR; A/T (rs7591)	0.40
	*AXIN2.2*	Intronic; C/T (rs4074947)	0.24
	*AXIN2.3*	Intronic; A/G (rs7224837)	0.10
	*AXIN2.4*	Intronic; A/T (rs11655966)	0.24
	*AXIN2.5*	Intronic; A/G (rs4128941)	0.04
	*AXIN2.6*	Intronic; A/G (rs11079571)	0.21
	*AXIN2.7*	Intronic; A/G (rs3923087)	0.27
	*AXIN2.8*	Intronic; G/T (rs3923086)	0.49
	*AXIN2.9*	coding, missense; A/G (rs2240308)	0.46

*BAX*	*BAX.1*	Intronic; C/T (rs1009316)	0.13
	*BAX.2*	Intronic; A/G (rs1805419)	0.27
	*BAX.3*	intronic; C/T (rs4645900)	0.05

*CTNNB1*	*CTNNB1.1*	promoter region; A/G (rs13075993)	0.41
	*CTNNB1.2*	Intronic; A/G (rs4135385)	0.25

*UGT1A1*	*UGT1A1*	Genomic, microsatellite; delTA (rs8175347)	0.34

*APC*	*APC.1*	Coding, missense; G/C (rs1801166)	0.01
	*APC.2*	Coding, missense ; A/T (rs459552)	0.21

*CARD15*	*CARD15.1*	Coding, missense; G/C (rs2066845)	0.02
	*CARD15.2*	Coding, insertion; ins C (rs5743293)	0.03

*MDR1*	*MDR1.1*	Coding, missense; G/T/A (rs2032582)	0.40, 0.02
	*MDR1.2*	Coding, silent; C/T (rs1045642)	0.50

*TS*	*TS.1*	3′UTR; delCTTTAA (rs16430)	0.31
	*TS.2*	Promoter region; 28-bp VNTR (2R/3R)	0.48

*HRAS1*	*HRAS1*	Genomic, minisatellite; 28-bp VNTR	0.19

**Table 2:** Association analysis between single polymorphisms and colorectal adenomas: *P*-values.

Polymophism	*P*-values for comparison*		
	SA versus PF	LA versus PF	SA versus LA**
*TGFBR2.1*	0.81	0.46	0.25
*TGFBR2.2*	0.66	0.63	0.86
*TGFBR2.3*	0.91	0.94	0.92
*MLH1.1*	0.87	0.16	0.30
*MLH1.2*	0.69	0.75	0.68
*MSH2.1*	0.33	0.87	0.32
*MSH2.2*	0.57	0.93	0.57
*MSH2.3*	0.64	0.88	0.55
*MSH2.4*	0.71	0.85	0.80
*MYH.1*	0.82	0.71	0.56
*P53.1*	0.56	0.23	0.20
*P53.2*	0.40	0.16	0.78
*P53.3*	0.16	0.41	0.86
*AXIN2.1*	0.13	0.66	0.76
*AXIN2.2*	0.78	0.63	0.49
*AXIN2.3*	0.29	0.46	0.85
*AXIN2.4*	0.19	0.08	0.75
*AXIN2.5*	0.37	0.60	0.27
*AXIN2.6*	0.77	0.55	0.48
*AXIN2.7*	0.27	0.18	0.06
*AXIN2.8*	0.38	0.29	0.53
*AXIN2.9*	0.53	**0.04**	**0.03**
*BAX.1*	0.95	0.68	0.80
*BAX.2*	0.95	0.26	0.48
*BAX.3*	0.64	0.49	0.83
*CTNNB1.1*	0.58	0.64	0.85
*CTNNB1.2*	0.93	0.15	0.48
*UGT1A1*	0.05	0.29	0.76
*APC.1*	0.17**	0.27***	0.04***
*APC.2*	0.80	0.26	0.38
*CARD15.1*	0.97	0.15	0.18
*CARD15.2*	0.48	0.57	0.97
*MDR1.1*	0.10	0.23	0.59
*MDR1.2*	0.69	**0.04**	0.09
*TS.1*	0.47	0.29	0.44
*TS .2*	0.41	0.25	0.87
*HRAS1*	0.38	0.17	0.65

*P-value for the Wald test assessing the global effect of SNP, uncorrected for multiple testing. **LA large adenoma, SA small adenoma, PF polyp-free. ***Exact test.

**Table 3:** Association analysis between single polymorphisms and colorectal adenomas.

Polymorphism	Genotype*	Odd ratio**		
		SA versus PF	LA versus PF	SA versus LA
*TGFBR2.1*	AC	0.91 [0.67−1.24]	1.06 [0.78−1.46]	1.22 [0.85−1.75]
	CC	0.91 [0.58−1.41]	1.31 [0.85−2.02]	1.51 [0.91−2.51]
*TGFBR2.2*	GC	1.07 [0.79−1.44]	0.95 [0.69−1.28]	0.91 [0.64−1.29]
	CC	1.23 [0.78−1.93]	1.18 [0.75−1.86]	0.91 [0.55−1.53]
*TGFBR2.3*	GC	0.93 [0.66−1.30]	1.00 [0.70−1.41]	1.08 [0.72−1.61]
	CC	0.94 [0.63−1.41]	1.06 [0.70−1.59]	1.09 [0.68−1.74]

*MLH1.1*	AG	1.08 [0.79−1.46]	1.01 [0.74−1.38]	0.90 [0.63−1.29]
	AA	1.11 [0.56−2.20]	1.80 [0.98−3.31]	1.64 [0.79−3.37]
*MLH1.2*	CT	1.04 [0.74−1.47]	1.14 [0.81−1.61]	1.08 [0.73−1.62]
	C	1.18 [0.79−1.78]	1.08 [0.71−1.64]	0.90 [0.55−1.46]

*MSH2.1*	AG	0.97 [0.72−1.31]	0.93 [0.69−1.27]	0.94 [0.66−1.34]
	GG	0.69 [0.43−1.13]	1.03 [0.65−1.62]	1.46 [0.83−2.54]
*MSH2.2*	AG	1.16 [0.86−1.57]	1.06 [0.78−1.44]	0.86 [0.60−1.23]
	GG	0.98 [0.61−1.57]	1.02 [0.64−1.62]	1.10 [0.64−1.89]
*MSH2.3*	GT	0.88 [0.65−1.90]	0.99 [0.73−1.35]	1.11 [0.78−1.58]
	GG	0.83 [0.45−1.53]	1.15 [0.65−2.04]	1.44 [0.72−2.85]
*MSH2.4*	CT	0.88 [0.65−1.20]	1.00 [0.74−1.37]	1.11 [0.77−1.58]
	CC	1.00 [0.53−1.90]	0.82 [0.40−1.65]	0.88 [0.40−1.94]

*MYH*	GC	1.05 [0.78−1.41]	0.89 [0.66-1.21]	0.85 [0.59−1.21]
	CC	0.86 [0.46−1.59]	1.06 [0.59-1.89]	1.15 [0.57−2.33]

*P53.1*	AG	0.95 [0.65−1.38]	1.37 [0.96−1.95]	1.43 [0.95−2.18]
	GG	0.50 [0.14−1.81]	1.01 [0.36−2.82]	1.62 [0.39−6.78]
*P53.2*	GC	1.17 [0.87-1.58]	1.34 [0.99−1-81]	1.11 [0.78-1.57]
	CC	1.35 [0.78−2.36]	1.25 [0.69−2.25]	0.91 [0.48−1.75]
*P53.3*	GC	1.25 [0.87−1.78]	1.13 [0.78−1.63]	0.91 [0.60−1.37]
	CC	0.40 [0.11−1.41]	0.53 [0.17−1.62]	1.28 [0.27−6.09]

*AXIN2.1*	AT	1.23 [0.89−1.69]	1.13 [0.82−1.56]	0.90 [0.62−1.31]
	AA	1.51 [1.01−2.27]	1.18 [0.78−1.79]	0.84 [0.53−1.35]
*AXIN2.2*	TC	1.06 [0.78−1.42]	0.95 [0.71−1.29]	0.93 [0.66−1.31]
	TT	1.23 [0.67−2.25]	0.72 [0.36−1.43]	0.64 [0.30−1.35]
*AXIN2.3*	AG	1.20 [0.84−1.71]	1.21 [0.84−1.73]	0.96 [0.64−1.44]
	GG	2.26 [0.64−8.01]	1.71 [0.45−6.52]	0.68 [0.18−2.66]
*AXIN2.4*	AT	1.22 [0.90−1.65]	1.40 [1.03−1.89]	1.13 [0.79−1.59]
	TT	1.51 [0.89−2.58]	1.35 [0.77−2.36]	0.95 [0.52−1.76]
*A* *X* *I* *N*2.5***	AG	1.26 [0.76−2.11]	0.86 [0.49−1.52]	0.70 [0.38−1.31]
*AXIN2.6*	AG	1.08 [0.79−1.46]	0.84 [0.62−1.15]	0.81 [0.57−1.16]
	AA	1.23 [0.63−2.38]	0.99 [0.49−1.99]	0.82 [0.38−1.79]
*AXIN2.7*	AG	1.18 [0.88−1.59]	0.76 [0.56−1.04]	0.66 [0.47−0.94]
	AA	1.48 [0.87−2.52]	1.07 [0.62−1.86]	0.74 [0.41−1.36]
*AXIN2.8*	GT	0.87 [0.62−1.23]	0.77 [0.55−1.08]	0.83 [0.56−1.24]
	GG	1.11 [0.74−1.66]	0.91 [0.61−1.37]	0.78 [0.49−1.24]
*AXIN2.9*	AG	1.15 [0.83−1.60]	1.34 [0.95−1.91]	1.22 [0.82−1.82]
	AA	0.96 [0.64−1.44]	1.67 [1.11−2.49]	1.86 [1.16−3.00]

*BAX.1*	TC	1.02 [0.73−1.42]	0.87 [0.61−1.22]	0.88 [0.59−1.29]
	TT	1.19 [0.38−3.72]	1.16 [0.37−3.65]	1.02 [0.28−3.74]
*BAX.2*	AG	0.97 [0.72−1.30]	0.83 [0.61−1.12]	0.87 [0.61−1.23]
	AA	1.05 [0.61−1.81]	1.23 [0.73−2.07]	1.24 [0.68−2.26]
*BAX*.3***	TC	0.89 [0.55−1.45]	0.73 [0.43−1.23]	0.83 [0.46−1.52]

*CTNNB1.1*	AG	1.14 [0.83−1.56]	1.03 [0.75−1.42]	0.90 [0.62−1.31]
	AA	1.23 [0.82−1.84]	1.21 [0.80−1.81]	0.93 [0.58−1.48]
*CTNNB1.2*	AG	1.12 [0.61−2.06]	1.41 [0.71−2.79]	1.22 [0.56−2.66]
	AA	1.13 [0.62−2.04]	1.73 [0.89−3.37]	1.44 [0.67−3.07]

*UGT1A1*	(TA)_6_/(TA)_7_	1.18 [0.86−1.62]	1.04 [0.76−1.41]	0.88 [0.62−1.26]
	(TA)_7_/(TA)_7_	0.63 [0.38−1.05]	0.70 [0.43−1.14]	1.00 [0.56−1.81]

*APC*.1***	GluGln	2.14 [0.67−7.00]	0.27 [0.01−2.11]	7.95 [1.05−354.36]
*APC.2*	AspVal	0.95 [0.70−1.28]	0.78 [0.57−1.05]	0.79 [0.56−1.14]
	ValVal	1.20 [0.58−2.49]	0.94 [0.43−2.06]	0.73 [0.31−1.72]

*CARD*15.1***	GlyArg	1.01 [0.49−2.09]	0.51 [0.20−1.29]	0.50 [0.18−1.38]
*CARD*15.2***	Ins C	0.78 [0.39−1.56]	0.68 [0.33−1.39]	0.89 [0.38−2.09]

*MDR1.1*	GT	0.89 [0.63−1.25]	0.88 [0.63−1.22]	0.92 [0.63−1.36]
	TT	1.32 [0.84−2.05]	1.15 [0.74−1.77]	0.91 [0.56−1.48]
	AG	0.60 [0.22−1.64]	0.29 [0.08−1.03]	0.46 [0.11−1.99]
	TA	2.86 [0.89−9.08]	1.27 [0.33−4.89]	0.41 [0.11−1.46]
*MDR1.2*	CT	1.00 [0.69−1.44]	0.76 [0.53−1.08]	0.69 [0.46−1.03]
	TT	1.16 [0.76−1.79]	1.16 [0.78−1.74]	1.01 [0.63−1.61]

*TS.1*	2R/1R	0.99 [0.73−1.36]	1.25 [0.92−1.71]	1.24 [0.87−1.77]
	1R/1R	1.35 [0.82−2.22]	1.33 [0.79−2.22]	0.99 [0.64−1.63]
*TS.2*	2R/3R	1.09 [0.75−1.59]	1.24 [0.85−1.82]	1.12 [0.72−1.75]
	3R/3R	1.31 [0.86−1.98]	1.43 [0.94−2.16]	1.10 [0.68−1.78]

*HRAS1*	rare alleles	1.16 [0.83−1.61]	1.26 [0.91−1.75]	1.09 [0.75−1.59]

*The genotype not shown is the reference one. **OR adjusted on age, sex, and group of centres; 95% CI is given into brackets. ***Very few individuals with the homozygous genotype were observed.

**Table:** (a) *P-*values of combinations of polymorphisms withthin single genes.

Genes	Haplotypic tests			Genotypic tests		
	PA versus PF	LA versus PF	LA versus SA	PA versus PF	LA versus PF	LA versus SA
*TGFBR2*	0.83	0.52	0.45	0.38	0.16	0.22
*MLH1*	0.64	0.41	0.63	0.45	0.24	0.13
*MSH2*	0.69	0.21	0.46	0.32	0.17	0.91
*TP53*	0.42	0.28	0.19	0.49	0.28	0.30
*AXIN2*	0.49	0.21	0.46	0.84	0.61	0.61
*BAX*	0.97	0.81	0.87	0.94	0.44	0.88
*CTNNB1*	0.43	0.16	0.37	0.79	0.43	0.76
*MDR1*	0.72	0.52	0.51	0.531	0.06	0.38
*APC*	0.16	0.26	**0.014**	0.19	0.45	0.08
*CARD15 *	0.61	0.28	0.40	0.67	0.27	0.41
*TS*	0.63	0.21	0.75	0.60	0.43	0.59

**Table:** (b) *P*-values of combinations of polymorphisms in different genes.

Genes groups	Haplotypic tests			Genotypic tests		
	PA versus PF	LA versus PF	LA versus SA	PA versus PF	LA versus PF	LA versus SA
*APC-MYH*	0.20	0.47	**0.045**	0.42	0.63	0.15
*CNNB1-HRAS*	0.47	0.25	0.58	0.74	0.31	0.58
*MDR1-TP53*	0.55	0.51	0.43	0.49	0.24	0.39
*AXIN2-APC*	0.36	0.19	0.07	0.19	0.73	0.40
*TP53-BAX-*						
*TGFBR2-APC*	0.19	0.42	0.26	0.64	0.52	0.67
*TS-MLH1-*						
*MSH2-MYH*	0.97	0.38	0.83	0.68	0.17	0.82
